# Widening of Socioeconomic Inequalities in U.S. Death Rates, 1993–2001

**DOI:** 10.1371/journal.pone.0002181

**Published:** 2008-05-14

**Authors:** Ahmedin Jemal, Elizabeth Ward, Robert N. Anderson, Taylor Murray, Michael J. Thun

**Affiliations:** 1 Epidemiology and Surveillance Research, American Cancer Society, Atlanta, Georgia, United States of America; 2 Division of Vital Statistics, National Center for Health Statistics, Centers for Disease Control and Prevention, Hyattsville, Maryland, United States of America; Copenhagen University Hospital, Denmark

## Abstract

**Background:**

Socioeconomic inequalities in death rates from all causes combined widened from 1960 until 1990 in the U.S., largely because cardiovascular death rates decreased more slowly in lower than in higher socioeconomic groups. However, no studies have examined trends in inequalities using recent US national data.

**Methodology/Principal Findings:**

We calculated annual age-standardized death rates from 1993–2001 for 25–64 year old non-Hispanic whites and blacks by level of education for all causes and for the seven most common causes of death using death certificate information from 43 states and Washington, D.C. Regression analysis was used to estimate annual percent change. The inequalities in all cause death rates between Americans with less than high school education and college graduates increased rapidly from 1993 to 2001 due to both significant decreases in mortality from all causes, heart disease, cancer, stroke, and other conditions in the most educated and lack of change or increases among the least educated. For white women, the all cause death rate increased significantly by 3.2 percent per year in the least educated and by 0.7 percent per year in high school graduates. The rate ratio (RR) comparing the least versus most educated increased from 2.9 (95% CI, 2.8–3.1) in 1993 to 4.4 (4.1–4.6) in 2001 among white men, from 2.1 (1.8–2.5) to 3.4 (2.9–3–9) in black men, and from 2.6 (2.4–2.7) to 3.8 (3.6–4.0) in white women.

**Conclusion:**

Socioeconomic inequalities in mortality are increasing rapidly due to continued progress by educated white and black men and white women, and stable or worsening trends among the least educated.

## Introduction

Socioeconomic inequalities in death rates from all causes combined have widened in the U.S.[1]–[7] since the early 1960s, largely because heart disease mortality has decreased more rapidly in higher than lower socioeconomic groups.[1], [3] However, most of the studies that used individual-level socioeconomic measures have been based on relatively small representative samples of the U.S. population rather than on national data, precluding simultaneous stratification by race, sex, and education in examining cause-specific trends. Further, no studies published in the peer-review literature have examined the trends since 1990 using national vital statistics. Such studies are important in measuring progress towards achieving the Healthy People 2010 objective of eliminating health disparities among subgroups of the population.

In this study, we examined mortality trends for all-causes and seven leading causes of death (cancer, heart disease, stroke, accidents, human immunodeficiency virus [HIV] infection, diabetes, and chronic obstructive pulmonary disease [COPD]) in relation to educational attainment from 1993 through 2001. These seven categories account for 70% of the total deaths. Mortality trends for several conditions decreased markedly over the past 10–15 years (cancer, heart disease, stroke, HIV infection) while others increased (accidents, diabetes, COPD), in part due to changes in risk factors and, for the former, advances in treatment.[8], [9] We restricted the analysis to white and black men and women, ages 25–64 years, an age range in which education is usually complete and provides a better index of socioeconomic position than at older ages.[10], [11]

## Methods

We obtained mortality data from 1993 through 2001 from the National Vital Statistics System (NVSS) administered by the National Center for Health Statistics. Underlying causes of death were classified according to the selection and coding rules of the Ninth Revision of the International Classification of Diseases (ICD-9)[12] for deaths recorded between 1993 and 1998, and according to the Tenth Revision of the International Classification of Diseases (ICD-10)[13] for those recorded between 1999 and 2001. The ICD-9 and ICD-10 codes for all causes and 15 leading causes included in this analysis are given on the web (Table S1).

Information on educational attainment (number of years of schooling) as a marker of socioeconomic position was obtained from death certificates. Although this information has been included on the standard death certificate since 1989, we restricted our analyses to the time interval 1993 to 2001 for three reasons: most states did not collect death certificate information on education systematically until 1993: race/ethnicity information collected by the Current Population Survey is not consistent with that of death certificates beginning with the 2002 survey (see below): and the question about educational attainment on the death certificate changed beginning with the 2003 mortality data from a highest grade of school completed to collegiate track education, i.e., the ability to identify degree status.[14] We classified educational attainment into four categories according to total years of schooling: less than high school graduate (<12 years of education), high school graduate (12 years), some college (13–15 years), and college graduate or post graduate (≥16 years).

We restricted the analyses to deaths among non-Hispanic whites and blacks (4,257,269), because of problems with reporting other racial and ethnic subgroups.[15] We excluded 609,093 deaths that occurred in seven states (Georgia, Kentucky, New York, Oklahoma, Rhode Island, South Dakota, and West Virginia) because completeness of education on death certificate in these states was less than 80% in at least one of the nine calendar years considered in this study. Another 126,238 deaths that occurred in the remaining 43 states and the District of Colombia were excluded due to missing data on education on the death certificates. The final analysis was based on 3,521,938 deaths recorded from 1993 to 2001; these comprised 97% of the total deaths among non-Hispanic whites and blacks in the 43 states and District of Colombia.

We obtained population data (the denominators for rate calculations) for the corresponding age, sex, race/ethnicity, education, state and time intervals from the U.S. Bureau of Census based on the Current Population Survey, a nationally representative sample of U.S. households.

Death rates (per 100,000) from all causes combined and from 15 leading causes were calculated by sex, race/ethnicity, educational attainment, and calendar year. Rates within the age range 25–64 years were age-standardized using the 2000 U.S. population standard. Temporal trends from all causes and seven selected cancer sites by educational attainment were assessed by fitting a weighted least squares regression models to the log-transformed annual age-standardized rates, weighted by the inverse of the variance, using joinpoint regression program, Version 3.0 software.[16] The annual percentage change or slope of the line segment was considered statistically significant if two-sided, P<0.05. Similarly, we also assessed the linearity of the annual percent changes across education levels within each race-, sex-, disease-specific category by fitting a weighted least square regression model to the log-transformed coefficients of the slopes, weighted by the inverse of the variance.[16] The educational disparity was assessed by calculating the ratio of the death rate for all causes combined in the least educated group (<12 years of education) to the most educated group (college degree or more) for 1993 and 2001. We also calculated the contribution of specific conditions to the overall decrease (in the case of heart disease, cancer, stroke, and HIV infection) or increase (diabetes, COPD, accidents) in mortality rates in the general population.[8], [17] This calculation was expressed as a percentage of the total change.

## Results

**Figure 1:**
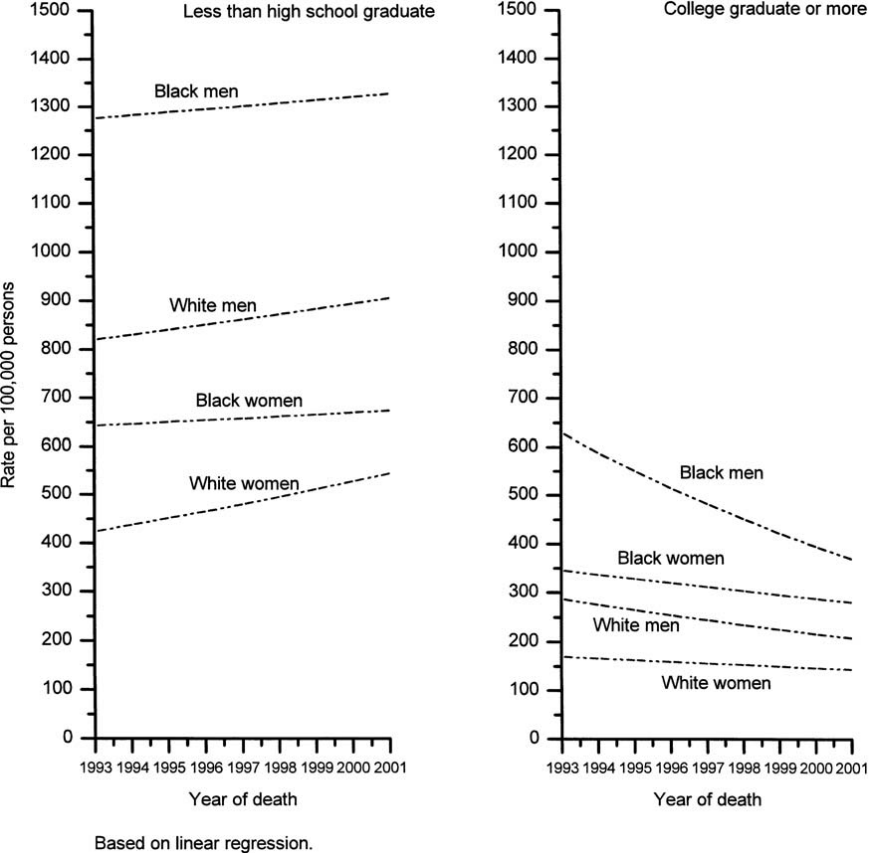
Temporal trends in age-standardized death rates from all causes combined among 25–64 year old U.S. adults by educational attainment, 1993–2001.

Table 1 and Figure 1 show trends in death rates from all causes combined from 1993 through 2001 among white and black men and women, 25–64 years old, in relation to educational attainment. The all cause death rate decreased significantly during this interval among the most educated (≥16 years) men and women, with the largest decrease in black men. In contrast, the all cause death rate increased in those with less than a high school education. The annual percent increase was largest among white women with less than 12 years of education (3.2% per year), but was also statistically significant (0.7% per year) in white women who had completed high school (Table 1). Between 1993 and 2001, the ratio of the all cause death rate in persons with <12 years versus ≥16 years of education increased from 2.9 (95% CI, 2.8–3.1) to 4.4 (4.1–4.6) in white men, from 2.1 (1.8–2.5) to 3.4 (2.9–3–9) in black men, from 2.6 (2.4–2.7) to 3.8 (3.6–4.0) in white women and from 1.8 (1.5–2.1) to 2.0 (1.8–2.3) over this nine-year interval. Among black women this ratio increased, but the trend was not statistically significant.

**Table 1:** Trends in age-standardized death rates (per 100,000) from all causes combined among 25–64 year old U.S. adults by race, sex, and education, 1993–2001

		Whites					Blacks				
		1993		2001			1993		2001		
Sex	Education in years	No. of Deaths	Rate	No. of Deaths	Rate	Annual %*	No. of Deaths	Rate	No. of Deaths	Rate	Annual %*
Men											
	All	197085	471.5	198728	414.9	−1.9†	49922	1019.3	49566	807.8	−3.2†
	<12 Years	46187	836.8	38638	931.1	1.3	18622	1253.5	14861	1283.1	0.5
	12 Years	83895	591.6	88435	596.0	−0.4	21553	1251.7	23722	1039.7	−2.6†
	13–15 Years	33479	348.9	37255	296.2	−2.3†	6310	631.5	7249	472.7	−4.0†
	16+ Years	33524	284.7	34400	212.7	−3.9†	3437	596.2	3734	381.6	−6.4†
	P value trend					0.001					0.008
	Rate difference (<12 vs. 16+)		552.2		718.4			657.3		901.5	
	Rate ratio, 95% CI (<12 vs. 16+)	2.9 (2.8–3.1)		4.4 (4.1–4.6)			2.1 (1.8–2.5)		3.4 (2.9–3.9)		
Women											
	All	110539	255.6	122950	247.3	−0.5†	29297	501.9	34341	476.7	−0.9†
	<12 Years	24473	422.4	21711	553.4	3.2†	9888	622.6	8614	622.3	0.6
	12 Years	53250	296.1	57153	321.8	0.7†	12688	612.9	15478	634.2	−0.5
	13–15 Years	18976	184.9	24865	177.7	−0.1	4375	331.1	6612	327.3	0.1
	16+ Years	13840	165.4	19221	146.1	−2.0†	2346	350.7	3637	308.2	−2.5†
	P value trend					0.01					0.26
	Rate difference (<12 vs. 16+)		257.1		407.3			272.0		314.2	
	Rate ratio, 95% CI (<12 vs. 16+)	2.6 (2.4–2.7)		3.8 (3.6–4.0)			1.8 (1.5–2.1)		2.0 (1.8–2.3)		

Rates were age standardized to the 2000 U.S. standard population.

* Annual percent change based on rates that were age-adjusted to the 2000 U.S. standard population using regression analysis.

† Annual percent change is statistically significantly different from zero (two-sided P<.05).

Among men, the temporal decrease in all cause death rates became larger with each additional increment of educational attainment. For example, the annual percentage decrease among black men was 2.6% for those with 12 years of education, 4.0% in men with 13–15 years of education, and 6.4% in those with ≥16 years of education. Among women, death rates from all causes combined decreased during this time interval only in black and white women with ≥16 years of education (Table 1).

Table 2 depicts trends in death rates from 1993 through 2001 for persons with <12 years of education and for those with ≥16 years of education for four major causes of death that are known to be decreasing in the general population. For all of these conditions, death rates decreased among the most educated. In contrast, the only condition for which the death rate decreased significantly among those with <12 years of education was HIV infection among white men. For white women with <12 years of education, the death rate from cancer increased by 1.1% per year and that from heart disease and stroke increased by 1.8% annually during the same time interval. The trends in death rates and the number of deaths in 1993 and 2001 for each race, sex, disease, and education specific group are provided in Tables S2 on the web.

**Table 2:** Trends in age-standardized death rates (per 100,000) for selected causes of death with decreasing trend in the general population among 25–64 year old U.S. adults by race, sex, and education, 1993–2001

		Whites			Blacks		
		Rate			Rate		
Cause/Sex	Education in years	1993	2001	Annual %*	1993	2001	Annual %*
Cancer							
Men	All	127.9	110.2	−1.9†	221.1	173.5	−2.7†
	<12 Yrs	195.3	208.3	0.8	256.0	246.8	0.1
	16+ Yrs	85.6	66.4	−3.1†	125.0	85.2	−5.1†
	Rate difference (<12 vs. 16+)	109.7	141.9		131.0	161.6	
	Rate Ratio, 95% CI (<12 vs. 16+)	2.3 (2.1–2.4)	3.1(2.9–3.4)		2.0 (1.6–2.6)	2.9 (2.4–3.5)	
Women	All	111.9	98.0	−1.7†	145.1	132.1	−1.1†
	<12 Yrs	142.0	157.7	1.1†	146.4	130.7	−0.3
	16+ Yrs	87.6	72.5	−2.7†	131.0	113.1	−2.6†
	Rate difference (<12 vs. 16+)	54.5	85.2		15.4	17.6	
	Rate Ratio, 95% CI (<12 vs. 16+)	1.6 (1.5–1.7)	2.2 (2.0–2.3)		1.1 (0.9–1.4)	1.2 (1.0–1.4)	
Heart Diseases							
Men	All	129.3	100.7	−3.2†	245.8	194.9	−2.7†
	<12 Yrs	228.8	214.9	−0.6	281.7	262.9	−0.2
	16+ Yrs	72.3	51.1	−4.2†	139.3	99.2	−5.1†
	Rate difference (<12 vs. 16+)	156.5	163.8		142.4	163.7	
	Rate Ratio, 95% CI (<12 vs. 16+)	3.2 (3.0–3.4)	4.2 (3.9–4.6)		2.0 (1.6–2.5)	2.6 (2.2–3.1)	
Women	All	44.8	37.9	−2.1†	122.0	106.1	−1.8†
	<12 Yrs	84.4	97.8	1.8†	151.4	132.9	−0.4
	16+ Yrs	20.0	16.9	−2.9†	73.1	62.8	−2.9†
	Rate difference (<12 vs. 16+)	64.4	80.9		78.3	70.1	
	Rate Ratio, 95% CI (<12 vs. 16+)	4.2 (3.9–4.6)	5.8 (5.3–6.3)		2.1 (1.7–2.6)	2.1 (1.8–2.5)	
Stroke							
Men	All	12.0	10.3	−1.9†	44.1	37.2	−2.8†
	<12 Yrs	22.4	23.9	1.0	55.3	55.8	−0.4
	16+ Yrs	6.5	5.0	−3.1†	21.4	18.8	−2.4†
	Rate difference (<12 vs. 16+)	15.9	18.9		33.9	37.0	
	Rate Ratio, 95% CI (<12 vs. 16+)	3.5 (3.1–3.9)	4.8 (4.3–5.4)		2.6 (1.9–3.5)	3.0 (2.4–3.8)	
Women	All	9.9	8.6	−1.8†	30.2	28.0	−1.4†
	<12 Yrs	15.9	19.2	1.8†	34.4	35.4	0.4
	16+ Yrs	5.9	4.6	−3.1†	21.5	15.7	−4.4†
	Rate difference (<12 vs. 16+)	10.1	14.6		12.8	19.7	
	Rate Ratio, 95% CI (<12 vs. 16+)	3.7 (2.4–3.1)	4.2 (3.7–4.7)		1.6 (1.2–2.1)	2.3 (1.8–2.8)	
HIV Infection							
Men	All	31.3	6.3	−23.0†	111.3	56.1	−12.1†
	<12 Yrs	28.7	15.4	−12.5†	123.1	120.9	−3.1
	16+ Yrs	31.4	3.5	−28.4†	118.2	28.9	−20.1†
	Rate difference (<12 vs. 16+)	−2.7	12.0		4.9	92.0	
	Rate Ratio, 95% CI (<12 vs. 16+)	0.9 (0.8–1.0)	4.5 (3.9–5.1)		1.0 (0.9–1.2)	4.2 (3.5–5.0)	
Women	All	1.9	0.9	−11.9†	23.1	21.7	−4.6
	<12 Yrs	5.7	5.4	−3.4	41.0	52.9	0.6
	16+ Yrs	0.8	0.1	−22.9†	8.9	5.9	−10.7†
	Rate difference (<12 vs. 16+)	4.9	5.2		32.2	47.1	
	Rate Ratio, 95% CI (<12 vs. 16+)	7.1 (5.2–9.5)	39.2 (22.4–68.6)		4.6 (3.2–6.8)	9.0 (6.3–12.9)	

Rates were age standardized to the 2000 U.S. standard population.

* Annual percent change based on rates that were age-adjusted to the 2000 U.S. standard population using regression analysis.

† Annual percent change is statistically significantly different from zero (two-sided P<.05).

Table 3 shows mortality trends among the least (<12 years of education) and most (≥16 years) educated persons by race and sex for three major conditions (accidents, diabetes, and COPD) known to have stable or increased death rates in the general population. Among the least educated, death rates increased for each of these conditions except for diabetes in black men and women for whom it did not change significantly. In contrast, among the most educated group, rates significantly decreased for COPD in white and black men and white women, for diabetes in black men and women and for accidents in black men, or remained stable for the remaining sex, race, and disease categories. The increases in death rates from 1993 through 2001 were inversely related to educational attainment (Table S3 on the web). For COPD, the death rate increased significantly among the least educated while decreasing significantly among men and women who had completed college in all sub groups except for black women for whom it did not change significantly.

**Table 3:** Trends in age-standardized death rates (per 100,000) for selected leading causes of death with increasing or leveling of trend in the general population among 25–64 year old U.S. adults by race, sex, and education, 1993–2001

		Whites			Blacks		
		Rate			Rate		
Cause/Sex	Education in years	1993	2001	Annual %*	1993	2001	Annual %*
Accidents							
Men	All	40.5	46.2	1.7†	69.4	63.8	−0.6
	<12 Yrs	93.2	119.9	3.4†	95.1	112.8	3.6†
	16+ Yrs	18.9	18.7	−0.10	31.7	27.6	−2.4†
	Rate difference (<12 vs. 16+)	74.4	101.2		63.4	85.2	
	Rate Ratio, 95% CI (<12 vs. 16+)	4.9 (4.6–5.3)	6.4 (6.0–6.8)		3.0 (2.5–3.7)	4.1 (3.4–4.9)	
Women	All	13.7	17.5	2.9†	18.1	20.9	1.2†
	<12 Yrs	28.7	47.2	6.7†	26.1	35.6	3.6†
	16+ Yrs	9.2	9.0	−0.30	10.9	10.8	−3.10
	Rate difference (<12 vs. 16+)	19.4	38.2		15.2	24.8	
	Rate Ratio, 95% CI (<12 vs. 16+)	3.1 (2.8–3.4)	5.3 (4.8–5.8)		2.4 (1.7–3.4)	3.3 (2.6–4.1)	
Diabetes							
Men	All	9.6	11.3	1.7†	27.5	29.6	0.70
	<12 Yrs	17.6	24.9	4.1†	31.1	38.4	3.2†
	16+ Yrs	4.9	5.1	0.70	17.8	16.0	−3.0†
	Rate difference (<12 vs. 16+)	12.7	19.8		13.3	22.4	
	Rate Ratio, 95% CI (<12 vs. 16+)	3.6 (3.2–4.1)	4.9 (4.4–5.4)		1.7 (1.2–2.6)	2.4 (1.9–3.1)	
Women	All	7.4	7.7	0.40	23.4	24.3	0.10
	<12 Yrs	14.1	20.0	4.0†	30.0	32.2	1.40
	16+ Yrs	3.6	3.3	−1.50	15.5	11.0	−4.3†
	Rate difference (<12 vs. 16+)	10.5	16.7		14.5	21.2	
	Rate Ratio, 95% CI (<12 vs. 16+)	3.9 (3.3–4.5)	6.0 (5.2–6.9)		1.9 (1.3–2.8)	2.9 (2.3–3.8)	
COPD							
Men	All	11.9	11.02	−0.70	16.9	14.5	−1.10
	<12 Yrs	26.6	34.7	3.6†	19.3	21.9	2.4†
	16+ Yrs	4.0	3.0	−3.9†	6.9	3.8	−5.7†
	Rate difference (<12 vs. 16+)	22.6	31.7		12.4	18.1	
	Rate Ratio, 95% CI (<12 vs. 16+)	6.7 (5.9–7.6)	11.7 (10.3–13.3)		2.8 (1.7–4.8)	5.8 (3.6–9.5)	
Women	All	10.1	10.4	0.50	10.6	10.8	0.40
	<12 Yrs	20.7	35.2	6.5†	12.8	15.7	2.4†
	16+ Yrs	3.6	3.1	−3.5†	4.4	5.4	1.60
	Rate difference (<12 vs. 16+)	17.1	32.1		8.4	10.3	
	Rate Ratio, 95% CI (<12 vs. 16+)	5.8 (5.0–6.8)	11.5 (10.0–13.3)		2.9 (1.7–5.0)	2.9 (1.9–4.3)	

Rates were age standardized to the 2000 U.S. standard population.

* Annual percent change based on rates that were age-adjusted to the 2000 U.S. standard population using regression analysis.

† Annual percent change is statistically significantly different from zero (two-sided P<.05).

In persons with <12 years of education, about 40% of the total increase in all cause death rates between 1993 and 2001 in white men was due to increases from accidents, cancer, and suicide, and nearly 50% of the total increase in white women was from accidents, cancer, COPD, and heart disease (Table S4 on web). In both white men and women, accidents contributed the largest percentage. In blacks, increases from accidents and nephritis in men and from accidents, HIV, and septicemia in women were offset by decreases from heart disease and cancer.

In persons with ≥16 years of education, about 90% of the total decrease in death rates from all causes between 1993 and 2001 among white men and 78% among black men were due to reduction in death rates from HIV infection, heart disease, and cancer, with HIV infection alone contributing over 50% in this age range (Table S4 on the web). In women with comparable education, the reduction in death rates from cancer, heart disease, and stroke during the corresponding time interval accounted for about 80% of the total decrease in all cause mortality rates in whites and 65% of the decrease in blacks. The reduction in death rates from cancer alone accounted for 65% and 35% of the total decrease in all-cause mortality among the most educated white and black women, respectively.

## Discussion

Our principal finding is that socioeconomic inequalities in mortality continue to increase in the U.S. due to reductions in death rates among the most educated combined with lack of progress or worsening trends in the least educated. As noted, the ratio of the all cause death rate in persons with <12 years versus ≥16 years of education increased from 2.9 (95% CI, 2.8–3.1) to 4.4 (4.1–4.6) in white men, from 2.1 (1.8–2.5) to 3.4 (2.9–3–9) in black men, and from 2.6 (2.4–2.7) to 3.8 (3.6–4.0) in white women. Contributing to the inequality is the unprecedented decrease in the all-cause death rate among the most educated men (totaling 36% in black men and 25% in white men over the nine-year interval) largely due to decreases in death rates from HIV infection, cancer, and heart disease.

Lower educational attainment, a marker of socioeconomic position, is associated with a host of environmental, social and economic factors that detrimentally affect health over a life time. People with less education have fewer financial resources, less access to health insurance or stable employment, and less health literacy. Social and economic factors increase the vulnerability of low socioeconomic communities to risk factors such as smoking, obesity, physical inactivity, hypertension, and HIV infection.[18]–[22] People without health insurance are less likely to receive basic preventive services or standard timely treatment.[23], [24] Those with lower health literacy are less likely to seek medical attention for asymptomatic conditions or to navigate the health care system effectively.[25], [26] The prevalence of most major risk factors remains much higher among persons with lower than high socioeconomic position.[27]–[29] Progress in reducing smoking has been slowest among the least educated.[27], [30] The prevalence of obesity, hypertension, and diabetes has increased in all socioeconomic position groups in recent years.[27], [30]–[33]

These environmental and socioeconomic factors may also have contributed to the differences in inequalities in all-cause and cause-specific mortality by race and sex. For example, the increases in death rates for cancer and heart disease among white women with <12 years of education but not among white men with <12 years of education partly reflects the later uptake of cigarette smoking among women than men. Smoking prevalence in women peaked about 20 years after it peaked in men.[34], [35] The large inequality in HIV infection mortality rates between blacks and whites may reflect an increased risk of HIV infection from sexual behavior and drug use as well as lower receipt of highly active antiretroviral therapy in blacks than in whites[36], [37]

Our findings are qualitatively similar to those of previous studies that analyzed temporal trends in US mortality by socioeconomic position in earlier time periods.[1], [3], [6] Using the 1960 National Mortality Follow Back Survey and 1986 National Health Interview Survey, Pappas et al documented that the disparity in mortality rates according to education increased from 1960 to 1986 among white and black, men and women, age 25–64 years, although the increase in disparity was less for women than men.[3] Feldman et al. reported widening of the educational disparity in death rates from 1960 to the time period 1971–1984 among white men 55 years or above, but not white women.[1] Similarly, using the National Occupational Mortality Surveillance, Steenland and colleagues reported that the differential in mortality by socioeconomic position increased from 1984 to 1997 among men age 35–64, but not in women.[6] In all of these studies, the disparity in death rates increased over time due to slower progress among those with lower than higher socioeconomic position. Our study, in contrast, finds no decrease in all cause mortality rates in the lowest educational group between 1993 and 2001. Furthermore, all-cause mortality rates among lower educated white women increased rather than decreased, with accidents, cancer, COPD, and heart disease accounting for about half of the total increase in white women with <12 years of education.

Mortality data collected after 2001 could not be analyzed using the same category of race/ethnicity. However, analysis of the 2005 mortality data by education for all races combined showed that the educational inequalities in all-cause mortality continued to be substantial.[38] Among 25–64 year olds, the all-cause mortality rate for those with <12 years of education was 3.2 times higher than that for persons with ≥13 years of education. The results of these studies illustrate the difficulty of achieving the goal of eliminating health disparities among disadvantaged subgroups of the population by 2010. Socioeconomic factors that contribute to poor nutrition, tobacco use, and physical inactivity often establish these patterns early in life[39]. Lower socioeconomic groups also have less access to preventive resources and health care. The elimination of health disparities will require addressing a broad spectrum of factors in the social and physical environment that contribute to poor health.

Strengths of our study include the use of national data that encompass 83% of the non-Hispanic white and black population in the U.S. from 1993 through 2001. The large number of deaths allows more precise statistical estimates of death rates by race and sex for specific causes of death. By limiting our analysis to broad disease categories for which death certificate data are more accurate,[40] we minimize inaccuracies in the cause of death.

Our major findings are not affected by coding changes implemented in ICD-10, beginning with the 1999 mortality data. These slightly accelerated the decline in death rates from heart disease and the increase from accidents, diabetes, and COPD (in women), and it may also have attenuated the decrease from cancer,[41] stroke, HIV infection,[42] and COPD (in men). ICD-10 allocated fewer deaths (1.5% of total) to heart disease as the underlying cause of death than ICD-9, but more deaths to the other causes. Increases in other causes in ICD-10 compared to ICD-9 ranged from 0.7% for cancer to 6.4% for HIV infection.[43] However, adjustments of rates using ICD comparability ratios for two of the leading causes most affected by the ICD changes (heart disease and HIV infection) had little effect on our findings. The annual percent decreases in heart disease death rates among white men with ≥16 years of education with and without adjustment for the change in ICD coding rules were, respectively, −4.0% (−4.5% : −3.5%) and −4.2% (95% CI, −4.7% : −3.7%). The corresponding annual percent decreases in HIV infection mortality rates among black men with <12 years of education were −3.9% (−9,9% : 2.4%) and −3.1% (−9.2%: 3.4%). Furthermore, the unfavorable mortality trends among less-educated men and women was observed for all causes of death combined, a category that is not affected by the change in disease classification, as well as for most of the seven specific causes described here.

A limitation of our study is that death certificates capture only one indicator of socioeconomic position, educational attainment. Socioeconomic position is a multi-factorial construct that reflects a combination of individual- and geographic area-level influences. Although it is optimal to consider multiple indices of socioeconomic position in examining relationships with health outcomes,[39], [44] educational attainment alone is frequently used as an indicator because of its availability, stability, and close association with other indicators of socioeconomic position for which data are not routinely available.[45]

Another limitation of our study is its reliance on years of education as reported on death certificates by next of kin. This variable has been included on the standard death certificate since 1989. Sorlie and Johnson evaluated this information in relation to self reported data from the National Longitudinal Mortality Study (NLMS) and found that education reported by proxies on the death certificate was higher than self-reported education in NLMS; 39% of adults who did not complete high school according to self-reported data were listed as high school graduates on their death certificates.[46] Although the extent of this misclassification may have varied over the study period (1993–2001), this particular source of error would have little impact on overall conclusions of our paper because the mortality trends are unfavorable in both groups of lower educated categories: 12 and fewer than 12 years of education.

Changes in autopsy rates over time could also potentially affect trends in cause-specific mortality rates because of the importance of the autopsy to the information on death certificates.[47] We cannot examine changes in autopsy rates by educational attainment for our study period (1993–2001) because NCHS stopped collecting information on whether an autopsy was performed or not in 1995 and resumed in 2003. We examined data for 1993 and 1994 and found that the percentage of deaths with autopsies were low in all groups and did not differ substantially by educational attainment. This percentage was 7%–8% among the lowest educated and 9%–10% among the most educated. In between, the rates range from 9%–11% with no discernable gradient. Data for 2003–2005 generally showed similar pattern, although the rates were slightly lower. Based on these data, changes in autopsy rates over time are unlikely to affect the all-cause mortality trend by educational attainment.

Finally, the generalizability of our findings to all age groups within the U.S. may be limited in that the educational gradient at ages 65 and above may be narrowed by universal access to health care through Medicare. However, deaths in ages 25–64 are of societal importance because they affect individuals and families most likely in the workforce, raising children and/or supporting other family members. The generalizability of our findings may also be affected slightly by exclusion of data from seven states with incomplete reporting of education on death certificates. The 3.5% of decedents with missing education information on their death certificates were excluded from the numerator, but not the denominator, of the study population (remaining 43 states and District of Colombia). The percentage of decedents with missing information on education was slightly higher in 1993 (4.7%) than in 2001 (2.8%). We assessed whether this might affect the interpretation of our trends. Analyses that measured the temporal trends without exclusion of deaths with missing education information slightly strengthened the decreasing trends for some causes and weakened the increasing mortality trends for others. However, none of these differences affected our main findings.

In conclusion, socioeconomic inequalities in mortality rates are increasing in the U.S. due to continuing reductions in death rates among the most educated white and black men and white women, but lack of progress or worsening trends in the least educated persons.

## Supporting Information

Table S1 International Classification of Diseases (ICD) codes for major causes of death according to the Tenth and Ninth Revisions
(0.03 MB XLS)

Table S2 Trends in age-standardized death rates (per 100,000) for selected causes of death with decreasing trend in the general populationamong 25–64 year old U.S. adults by race, sex, and education, 1993–2001
(0.04 MB XLS)

Table S3 Trends in age-standardized death rates (per 100,000) for selected leading causes of death with increasing or leveling of trend in the general population among 25–64 year old U.S. adults by race, sex, and education, 1993–2001
(0.04 MB XLS)

Table S4 The contributions of specific causes to the total increase (in less educated persons) or decrease (in most educated persons)in the all cause mortality rate (per 100,000) between 1993 and 2001 in 25–64 year old U.S. adults by race and sex
(0.04 MB XLS)
